# Active *in vivo* translocation of the *Methanosarcina mazei* Gö1 Casposon

**DOI:** 10.1093/nar/gkad474

**Published:** 2023-05-31

**Authors:** Finn O Gehlert, Lisa Nickel, Nikolaos Vakirlis, Katrin Hammerschmidt, Herman I Vargas Gebauer, Claudia Kießling, Anne Kupczok, Ruth A Schmitz

**Affiliations:** Institute for General Microbiology, Christian Albrechts University, 24118Kiel, Germany; Institute for General Microbiology, Christian Albrechts University, 24118Kiel, Germany; Institute for General Microbiology, Christian Albrechts University, 24118Kiel, Germany; Institute for Fundamental Biomedical Research, Biomedical Sciences Research Center ‘‘Alexander Fleming’’, Vari, Greece; Institute for General Microbiology, Christian Albrechts University, 24118Kiel, Germany; Institute for General Microbiology, Christian Albrechts University, 24118Kiel, Germany; Institute for General Microbiology, Christian Albrechts University, 24118Kiel, Germany; Institute for General Microbiology, Christian Albrechts University, 24118Kiel, Germany; Bioinformatics Group, Wageningen University & Research, 6708PBWageningen, Netherlands; Institute for General Microbiology, Christian Albrechts University, 24118Kiel, Germany

## Abstract

Casposons are transposable elements containing the CRISPR associated gene Cas1solo. Identified in many archaeal genomes, casposons are discussed as the origin of CRISPR-Cas systems due to their proposed Cas1solo-dependent translocation. However, apart from bioinformatic approaches and the demonstration of Cas1solo integrase and endonuclease activity *in vitro*, casposon transposition has not yet been shown *in vivo*. Here, we report on active casposon translocations in *Methanosarcina mazei* Gö1 using two independent experimental approaches. First, mini-casposons, consisting of a R6K**γ** origin and two antibiotic resistance cassettes, flanked by target site duplications (TSDs) and terminal inverted repeats (TIRs), were generated, and shown to actively translocate from a suicide plasmid and integrate into the chromosomal MetMaz-C1 TSD IS1a. Second, casposon excision activity was confirmed in a long-term evolution experiment using a Cas1solo overexpression strain in comparison to an empty vector control under four different treatments (native, high temperature, high salt, mitomycin C) to study stress-induced translocation. Analysis of genomic DNA using a nested qPCR approach provided clear evidence of casposon activity in single cells and revealed significantly different casposon excision frequencies between treatments and strains. Our results, providing the first experimental evidence for *in vivo* casposon activity are summarized in a modified hypothetical translocation model.

## Graphical Abstract

**Graphical Abstract:**
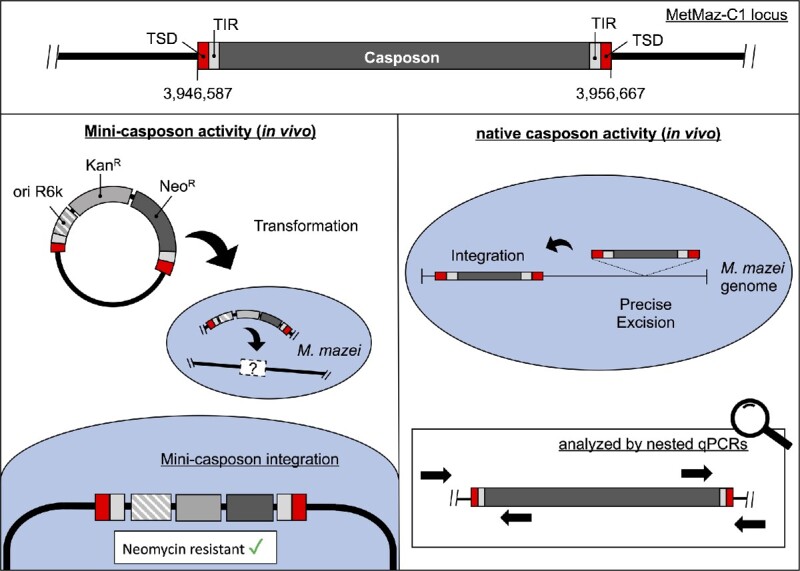

## INTRODUCTION

Transposons are mobile genetic elements (MGEs), found in the genomes of organisms of all domains of life. They occur in high quantities in plants, but are also present in animals, bacteria, and archaea (1–4). In general, MGEs are one major factor of evolution and adaptation, since their translocation within chromosomal and extra-chromosomal DNA can result in all kinds of mutations from large scale chromosomal rearrangements to single nucleotide polymorphisms (SNPs), often leading to gene silencing or generation of new coding genes (5,6). MGEs include all kinds of elements that can change their genomic position within a host genome or between host cells, including viruses, plasmids and transposons. Transposons are classified by their translocation mechanism - Class I comprising retrotransposons and Class II DNA transposons - and are further distinguished by their length and gene content (7–9). Class II transposons, which translocate primarily via a cut-and-paste mechanism (8,9), include both short insertion sequence (IS) elements, which often encode for only one transposase gene (e.g. Tc1 mariner superfamily elements) and large elements longer than 10 kb (e.g. Mavericks/Polintons) (8,10–12).

**Figure 1.**
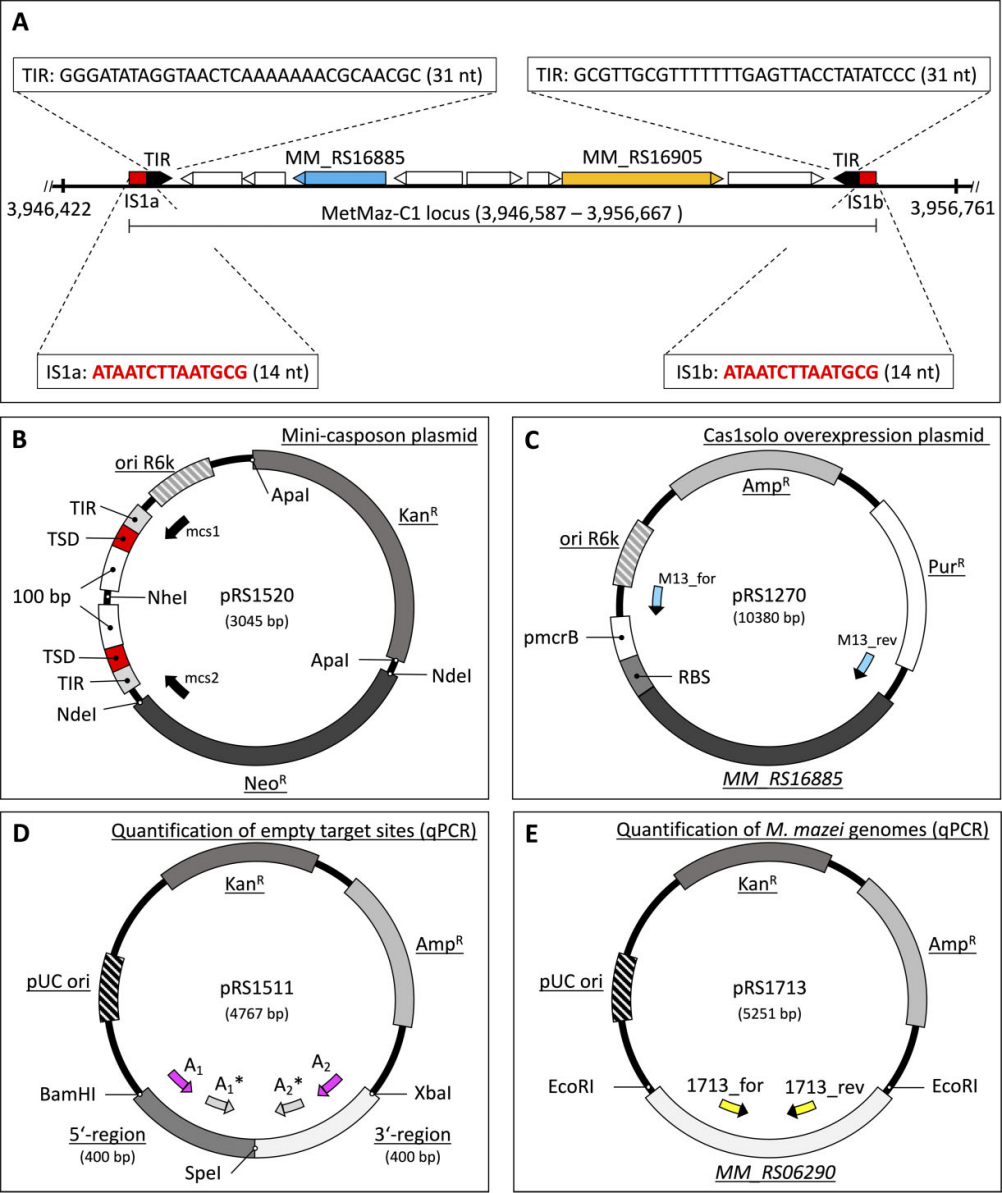
Overview of the native MetMaz-C1 locus and plasmids generated in this study. (**A**) Section of the *M. mazei* genome carrying the casposon MetMaz-C1 between genome positions 3946587 and 3956667 based on Krupovic and colleagues (17,19). MetMaz-C1 encodes 8 genes, highlighted are the genes encoding the key enzyme Cas1solo (*MM_RS16885*; blue) and the type B Polymerase (*MM_RS16905*; orange). (**B**) pRS1520 (mini-casposon on suicide plasmid) consisting of the MetMaz-C1 target site duplications (TSDs; red; 5-ATAATCTTAATGCG-3) and terminal inverted repeats (TIRs; light gray; 5-GGGATATAGGTAACTCAAAAAAACGCAACGC-3), flanking a R6K **γ** origin (gray striped), a kanamycin (gray) and neomycin resistance cassette (dark gray). Sequencing primers mcs1 and mcs2 used for testing of mini-casposon integration based on rescue cloning are depicted (black arrows). (**C**) pRS1270, Cas1solo overexpression plasmid with the native *cas1solo* gene (*MM_RS16885*) under the control of the constitutive promotor mcrB (pmcrB) and the respective ribosome binding site (RBS) in a pWM321 backbone (26). Sequencing primers (M13 for/rev; light blue) were used for cross contamination tests in the long-term evolution experiment. (**D**) pRS1511, based on the pCRII vector (TOPO™ TA Cloning™ Kit, Thermo Fisher Scientific, Waltham, MA, USA), was used as a control and for the normalization in casposon excision frequency (CEF) determination by nested qPCRs. The upstream (5′-region) and downstream regions of the native casposon MetMaz-C1 (3′-region) were PCR amplified and cloned into pCRII by restriction (using BamHI/XbaI). Primer sequences for the nested qPCRs are depicted as follows: primer set A (magenta; 1^st^ PCR) and primer set A* (light gray; 2^nd^ PCR). (**E**) pRS1713 used to determine the genome copy numbers for normalization of casposon excisions per genome. pRS1713 was generated by TOPO-TA cloning of *MM_RS06290* (bifunctional hexulose-6-phosphate synthase) into pCRII (TOPO™ TA Cloning™ Kit, Thermo Fisher Scientific, Waltham, MA, USA). qPCR primers, 1713_for and 1713_rev, targeting the introduced gene are depicted in yellow.

The distribution of MGEs in the archaeal domain is very uneven, since most of the known transposable elements are restricted to the orders of *Halobacteriales*, *Sulfolobales*, *Thermoplasmatales* and *Methanosarcinales* (3). *Methanosarcinales*, particularly *Methanosarcina mazei*, have been shown to carry a large number of transposable elements represented by 102 identified transposase encoding genes (mostly IS elements) (13). Within the last two decades, transposable elements were shown to be connected to adaptive immunity of eukaryotes and prokaryotes. In eukaryotes the key enzyme of V(D)J recombination (RAG1) was found to be derived from a *Transib* transposon (14,15). In contrast to this, clustered regularly interspaced short palindromic repeats (CRISPR) with CRISPR associated (Cas) proteins and complexes are representatives of an adaptive defense system in prokaryotes, with the ability to incorporate so-called spacer sequences derived from invader genomes into CRISPR arrays for future defense of the same invader by targeted degradation (reviewed in (16)). CRISPR derived immunity, with its three phases of targeted defense - adaptation, expression and interference - is completely dependent on expression of Cas proteins (reviewed in (16)). With the discovery of many different CRISPR-Cas systems showing clear homologies in functions and mechanisms, the question regarding the evolutionary origin of those systems has become a focus of attention. Solitary family members of the key CRISPR adaptation protein Cas1 were found on transposon-like elements, hence designated casposons, which led to the proposal that transposons contributed to the origin of CRISPR-Cas systems (17). While transposases are essential for the activity of typical transposable elements, this type of enzyme has not been found to be encoded in casposons. The only encoded enzyme common to all discovered casposons is Cas1solo or the so-called casposase, which was proposed to mediate the casposon translocation (17). This new class of DNA transposons has been proposed to be the first self-synthesizing member of DNA transposons in prokaryotes and bears some similarity to Polintons/Mavericks, due to the common type B polymerases in addition to their enormous size of several kilobase pairs (10,11,17). To date, four distinct casposon families are described and classified mainly by the domain structure of their Cas1solo enzyme (18,19). Krupovic and colleagues proposed a casposon translocation model close to the transposition of Polintons/Mavericks (11,17). They predicted a TIR mediated looping out of the casposon from the lagging strand during cellular DNA replication, followed by Cas1solo mediated excision, polB replication and Cas1solo mediated reintegration into the genome (17,20,21). The reintegration by generation of target site duplications was proposed to be very similar to the functions of the spacer adaptation module of the CRISPR-systems (17). Krupovic *et al.* were predicting a persistence of the target site duplication post excision (17). Although casposons were discovered almost ten years ago, final proof of principle is still lacking. Characterizations of casposon functions have been limited to *in vitro* assays, mainly based on heterologously expressed Cas1solo proteins analyzed for specific activities (22–25). Extensive studies of the casposon identified in *Aciduliprofundum boonei*, especially biochemically characterizing its Cas1solo variant, gave first insights into the translocation mechanism *in vitro* and suggested casposon activity *in vivo* (23–25). Various experiments with purified heterologously expressed Cas1solo of *A. boonei* and *M. mazei* showed that the enzymes were able to integrate substrates like casposon derivates or short synthetic oligonucleotides in a site-specific process into different target sites present on pUC19 derived acceptor plasmids (19,22–24). The site-specific integration showed indeed similarities to the integration of new spacer sequences into CRISPR arrays during the adaptation step of the CRISPR-Cas immunity process (17,22). To date, all studies conducted on casposons have lacked *in vivo* proof of principle. This is due to the fact that convenient model systems are exceedingly rare, as the number of casposon carrying host organisms, mostly archaeal species, which are culturable in laboratories is still very low as is the number of genetically tractable systems (17,19). Consequently, the aim of the here presented study was to detect *in vivo* activity of the *M. mazei* casposon MetMaz-C1 first described by Krupovic and colleagues (19). MetMaz-C1 has a size of approximately 10 kb, encodes eight genes and is flanked by a TIR of 31 nt (GGGATATAGGTAACTCAAAAAAACGCAACGC) and TSD of 14 nt (5-ATAATCTTAATGCG-3) ((17,19); Figure 1A).

Here, we report on the casposon MetMaz-C1 being an active MGE. We were able to characterize and quantify its excision frequency *in vivo*. For investigation of the casposon activity we established and used an optimized *in vivo* mini-casposon assay in *M. mazei Gö1* based on preceding *in vitro* studies (22–25). Our results show that MetMaz-C1 can excise by leaving an empty target site and actively integrates into new genomic loci or forms tandem structures by integrating into the original target sites. Based on our findings, we suggest to modify the previous model for translocation predicted by Krupovic and colleagues (17).

## MATERIALS AND METHODS

### Description of plasmids used in this study

All cloning strategies in this study were based on restriction cloning, for which all major restriction enzymes were purchased from New England Biolabs (NEB, Ipswich, MA, USA). In addition, all chemicals used in this study were purchased from Carl Roth GmbH + Co. KG (Karlsruhe, Germany) unless otherwise stated.

### Cloning of mini-casposon and cas1solo overexpression plasmid

The mini-casposon suicide vector (pRS1520) was generated based on the gene synthesis of the construct TSD-TIR-NdeI-ApaI-R6K **γ** origin-TIR-TSD inserted in a pEX-A256 backbone obtained from Eurofins Genomics Germany GmbH (Ebersberg, Germany). The kanamycin resistance cassette was cloned into the ApaI site. The mini-casposon sequence was amplified by PCR using LongAmp® Taq DNA Polymerase (NEB) according to manufacturer's protocol for deletion of the irrelevant pEX-A256 backbone. Both primer sequences Minicas_SDM_for and Minicas_SDM_rev (Supplementary Table S1) including terminal NheI restriction sites were binding to the plasmid upstream or downstream of the mini-casposon. Primers were annealed to the template at 68°C for 30 s, followed by elongation of the PCR products at 65°C for 5 min and a total of 35 cycles. *E. coli* DH5*α* cells were transformed with the resulting 2 kb PCR products, which were self-ligated post NheI restriction. The neomycin resistance cassette was PCR amplified from pRS830 using the primer set NeoR_for and NeoR_rev (Supplementary Table S1) and cloned into the NdeI site resulting in the final mini-casposon suicide vector pRS1520 (Figure 1B). Two oligonucleotides were designed to verify positive translocation into the *M. mazei* genome and to exclude integration of the complete plasmid into the chromosome: mini-casposon sequencing primers mcs1 and mcs2 (Figure 1B; Supplementary Table S1).

**Figure 2.**
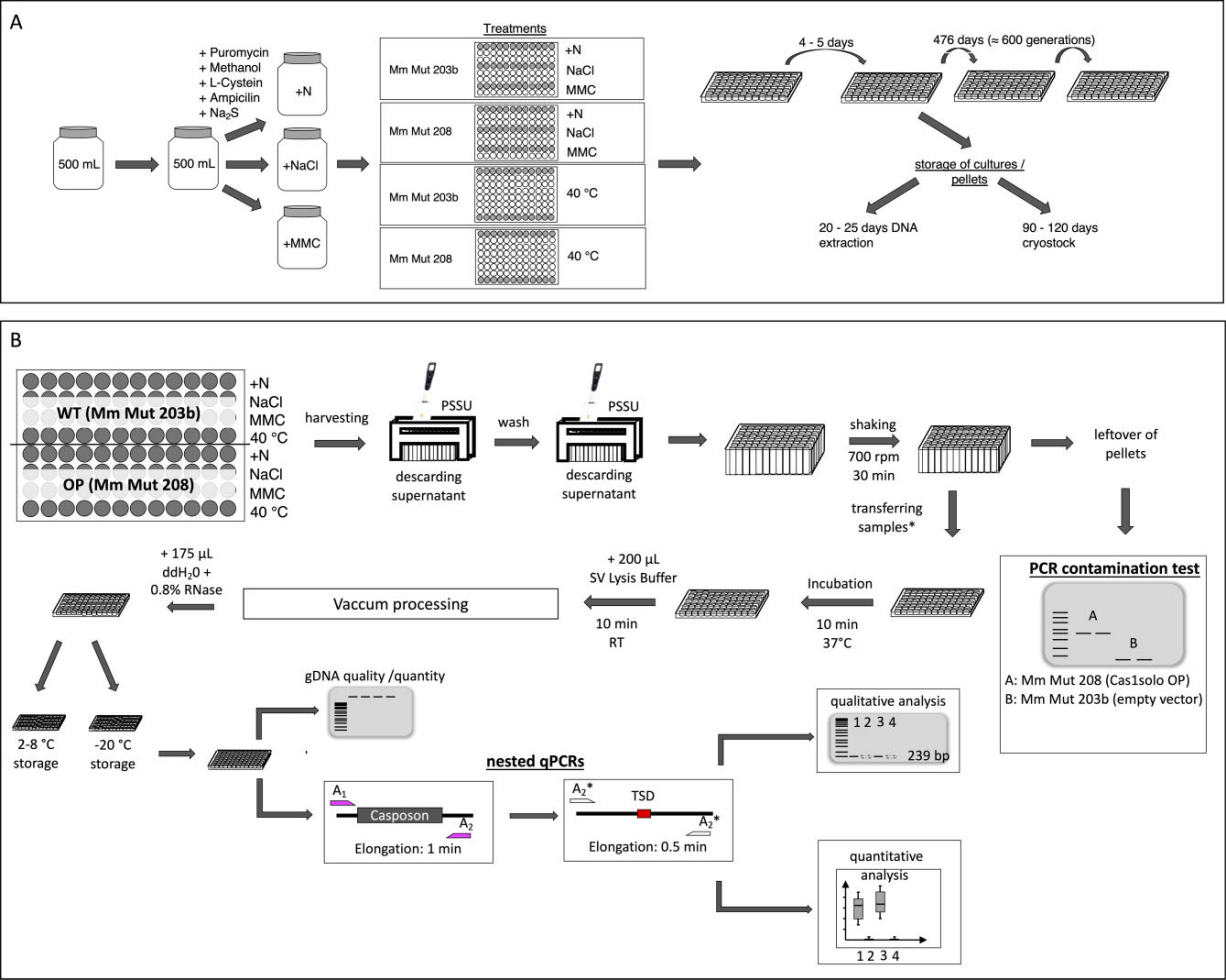
Set-up of the long-term evolution experiment. (**A**) Prepared minimal medium was split and complemented according to the respective treatment (500 mM NaCl, 0.5 μg/ml mitomycin C, 40°C, untreated controls (native; +N)). 1.4 ml respective medium was filled in used wells (gray) of 96-deep-well plates according to depicted pipetting scheme to reduce contaminations between treatments. Strains Mm Mut 203b, carrying an empty pWM321 vector, and Mm Mut 208, carrying the Cas1solo overexpression plasmid pRS1270, were cultivated under anaerobic conditions on separate deep-well plates, to exclude cross contamination. Medium was inoculated by transferring 50–200 μl of preceding cultures under consideration of roughly estimated cell densities. Plates were incubated for 4–5 days under appropriate treatment conditions (37°C or 40°C) and used for inoculations of the next round of plates. Cells were harvested by centrifugation for DNA isolation or were mixed with glycerol for cryo-storage at − 80°C. The long-term evolution experiment was running for 472 days representing a minimum of approximately 600 generations. (**B**) Post inoculation of new cultures, genomic DNA was isolated from the remaining cultures using the Wizard® SV 96 Genomic DNA Purification System (Promega, Madison, WI, USA) as described in the method section. DNA quality and quantity were verified by agarose gel electrophoresis. Casposon activity based on casposon excision frequency (CEF) was tested by nested qPCR reactions targeting flanking chromosome regions. PCR fragments were only generated in case of an empty casposon locus, due to short elongation times (0.5–1 min) and MetMaz-C1 size (approx. 10 kb) resulting in a final PCR product of 239 bp. Primer set A was used for the 1^st^ PCR, Primer set A* for the 2nd PCR (Figures 1D and 3). Quantification was performed under consideration of genome copy numbers in qPCR reactions.

Constitutive Cas1solo overexpression plasmid (pRS1270, Cas1solo OP, Figure 1C) was generated based on PCR amplification of *MM_RS16885* (*cas1solo*) using the primer set MM_RS16885_for and MM_RS16885_rev (Supplementary Table S1), and genomic DNA (gDNA) of *M. mazei* as PCR template. The PCR product (1236 bp) was cloned via NcoI and KpnI restriction into pRS1248 (pDRIVE backbone) under the control of the mcrB promotor (pmcrB) and with the addition of the mcrB ribosome binding site (RBS). Constructed fragments of pmcrB - RBS - *cas1solo* were cloned into the shuttle vector pWM321 (26) using KpnI and SacI, resulting in pRS1270 (Figure 1C). During this study *M. mazei* mutants were generated following the liposome-mediated transformation protocol of Ehlers and colleagues (27). Mutant strains were stably transformed with either pRS1270 (Mm Mut 208) or the empty pWM321 vector (Mm Mut 203b) as a control. Both strains were used side by side in a long-term evolution experiment, which required checking for cross contaminations between the strains. For this purpose, PCR contamination tests of subsamples with M13 primers (for/rev) were performed (Figures 1C and 2B). The PCR products were clearly separatable by gel electrophoresis. PCR fragments derived from the empty vector control were approx. 450 bp in size, while the PCR product of pRS1270 was roughly 1800 bp.

### Cloning of quantitative (q) PCR control plasmids pRS1511 and pRS1713

In the current study optimized nested PCR and qPCR protocols were established using plasmids as PCR controls or in case of qPCRs a plasmid normalization for absolute quantification. For this purpose, two different control plasmids were generated.

pRS1511 was designed as a PCR control and for normalization of nested qPCRs to determine the casposon excision frequency (CEF). Two PCR products with terminal restriction sites and a length of 400 bp each of the upstream and downstream regions of the native casposon MetMaz-C1 were generated. The first 400 bp fragment was amplified by using the primers US_BamHI_for and US_SpeI_rev (Supplementary Table S1), and *M. mazei* gDNA. The second PCR fragment was generated by using the same template with primers DS_SpeI_for and DS_XbaI_rev (Supplementary Table S1). The first PCR fragment was digested by BamHI and SpeI and the second fragment by SpeI and XbaI. Both fragments were cloned to BamHI and XbaI linearized pCRII vector (Figure 1D). The final plasmid (pRS1511) was used in the nested qPCRs for normalization by standard curve. The plasmid was targeted with the two primer sets A_1_:A_2_ and A_1_*:A_2_* (Figure 1D; Supplementary Table S1).

The second qPCR normalization plasmid (pRS1713) was also based on the pCRII-TOPO vector but was used for the estimation of genome copy numbers for normalization to genome numbers in qPCR reactions. The house-keeping gene *MM_RS06290* encoding the bifunctional hexulose-6-phosphate synthase was PCR amplified from *M. mazei* gDNA using MM_RS06290_for and MM_RS06290_rev (Supplementary Table S1) and TA cloned to pCRII (Figure 1E). Primers 1713_for and 1713_rev primers targeting *MM_RS06290* were designed for qPCR reactions (Figure 1E). All primers used for cloning purpose or for the characterization of casposon activity are listed in Supplementary Table S1.

### Mini-casposon *in vivo* assay and rescue cloning

For investigation of the mini-casposon translocation in a plasmid-based assay, *M. mazei* Gö1 wildtype (DSM3647) was transformed with the generated pRS1520 suicide vector carrying the mini-casposon by liposome-mediated transformation (Figure 1B) (27). Optimizations, differing from the published methods of Ehlers and colleagues (27) are highlighted in the following. 50 ml log phase growing *M. mazei* culture was harvested by centrifugation for 10 min at 3.000 x g in oxygen free atmosphere and carefully resuspended in sucrose buffer (10 mM MES, 0.15 M sucrose, 6.3 pH). 1 μg pRS1520 was diluted in 50 μl sucrose buffer and added to mixture of 30 μl DOTAP (Roche Holding AG, Basel, Switzerland) and 70 μl sucrose buffer, followed by an incubation for > 30 min at 37°C. 990 μl of resuspended cells were added to sucrose-DNA-DOTAP mixture. Liposome-mediated transformation reactions were incubated for 5 h under anaerobic conditions and supplemented with 5 mM MgCl_2_ and 5 mM MnCl_2_ after 3 h. The cells were split to roughly 500 μl per reaction and transferred to 5 ml complemented minimal medium containing additionally 0.1 M sucrose. Cells were incubated over night at 37°C. New media containing neomycin (22 μM) were inoculated by transferring 500 μl overnight cultures. Cultures were incubated for 14–21 days until cell growth could be detected. Growing populations were transferred to new media immediately and reinoculated several times until gDNA for mini-casposon translocation analysis was isolated using Wizard Kit (Promega, Madison, WI, USA) as recommended. gDNA was dissolved in RNAse free water. Purified gDNAs were first analyzed by PCR using mcs1 and mcs2 primers to exclude potential false positives resulting from single-crossover events of pRS1520 (Figure 1B) with *M. mazei* genome e.g. by homologous sequences (e.g. IS-elements).

Populations with no detectable PCR fragments from the vector backbone were further analyzed for the presence of the mini-casposon by rescue cloning. For this purpose, 1 μg of isolated gDNA was restriction digested by AccI to completeness in a total volume of 50 μl using 20 U enzyme at 37°C for 2 h, followed by heat inactivation at 80°C for 20 min. Reactions were incubated on ice for 5 min and split into three T4 ligation reactions. T4 ligation reactions had a final volume of 20 μl with 0.1 U/μl ligase and were incubated for 1 h at room temperature (RT) prior transformation in chemical competent One Shot™ PIR1 (Thermo Fisher Scientific, Waltham, MA, USA) and selection for kanamycin resistance. Single clones were selected and sequenced via Sanger sequencing technology using mcs1 and mcs2 for sequencing out of the mini-casposon part into the potential genomic insertion site.

### Long-term evolution experiment - strain generation, growth conditions and setup

Single clones of *M. mazei* transformed with pRS1270 (Cas1solo OP, Figure 1C) or pWM321 (empty vector control, (26)) were generated like described (27). A single colony of *M. mazei* carrying pRS1270 was named Mm Mut 208, whereas empty vector control (pWM321) was designated Mm Mut 203b. Both strains were grown in an oxygen free atmosphere in 96-deep-well plates with 1.4 ml minimal medium per well (see Figure 2A) according to the methods adapted from (28,29). Sterile deep-well plates were stored in a sterile anaerobic chamber for three weeks prior usage in the experiment and were further ‘pre-reduced’ with 1 ml of sterile pre-reducing solution (PRS: 6.3 mM Na_2_S, 18.6 mM cysteine, 7.5 mM NaOH) per well for 2 h, followed by decanting and drying for 30 min. Minimal medium was flushed with sterile N_2_:CO_2_ (80:20) for 10 min and supplemented with methanol (150 mM), acetate (40 mM), Na_2_S (1 mM), cysteine (2 mM), puromycin dihydrochloride (9 μM; Merck, Darmstadt, Germany) and ampicillin (0.3 mM) or kanamycin sulfate (52 μM) as described before ((30)); Figure 2A). The bacteria-targeting antibiotics - kanamycin and ampicillin - were used alternating every second inoculation. According to the treatments, medium was either complemented with 500 mM NaCl (sodium chloride treatment) or 0.5 μg/ml mitomycin C (mitomycin treatment; Fisher Scientific GmbH, Schwerte, Germany), or was incubated at 40°C (temperature treatment) or left untreated (native; +N) (Figure 2A). The strains were split to different plates and treatments (+N; NaCl treatment; mitomycin treatment) were separated by two empty rows (Figure 2A). Temperature treated cultures were inoculated on a separate 96-deep-well plate. 12 replicates per strain and treatment were inoculated with 50–200 μl corresponding preceding culture (Figure 2A). The culture plates were closed with needle perforated silicon covers and custom-made autoclavable aluminum pressure plates (AAPP) to avoid evaporation and contamination. Prepared cultures were incubated for 4–5 days at 37°C (+N; NaCl; mitomycin) in an anaerobic chamber or in an anaerobic pot at 40°C (temperature treatment) with the same gas atmosphere (N_2_:CO_2_/80:20) until the next set of respective media was inoculated. After inoculating the next cultures, the remaining cultures were used to isolate gDNA and occasionally to generate a cryo-storage by storing mixtures of 60 μl of the respective culture and 25 μl sterile anaerobic glycerol (86%) in 96-microtiter plates at − 80°C (Figure 2A). The long-term evolution experiment ran for 472 days, which corresponds to approximately 600 generations by interpolating the lowest doubling time. The doubling time ranged around 16.5 ± 2.5 h for the different treatments (OD_600_ measurements and doubling time calculations can be found in the supplement: Supplementary Table S2).

For gDNA isolation, all cultures were transferred together to one single 96-deep-well plate and harvested by centrifugation at 3220 x g and 4°C for 20 – 30 min (Figure 2B). Since *M. mazei* cell pellets are not solid but rather mucoid, the supernatant was carefully discarded by pipetting with a self-developed 3D-printed pellet saving spacer unit (PSSU) (Figure 2B). Pellets were washed once with 1 ml of phosphate buffered saline (PBS: 0.73 M NaCl, 0.31 M Na_2_HPO_4_, pH 7.5), followed by a second centrifugation step. Supernatant was again discarded by pipetting using the PSSU (Figure 2B). Pellets were carefully resuspended in the remaining PBS volume of roughly 50 μl by shaking at 500 rpm for 20 min and stored at −20°C in microtiter plates or immediately processed with Wizard® SV 96 Genomic DNA Purification System (Promega) using Vac-Man® 96 Vacuum Manifold (Promega). Manufacturer protocol was adjusted according to cell pellet size and cell pellet morphology. Pellets were thawed for 10 min, carefully resuspended in 200 μl SV-Lysis buffer to avoid foaming and incubated at RT for 10 min. 275 μl mixture was transferred to the binding plate. After binding, washing and drying, gDNA was eluted with 175 μl water (0.8 % RNAse) and incubated for 10 min at RT. 60 μl aliquots of elutions were stored at 4°C and −20°C (Figure 2B).

For quality assessment culture plates were PCR screened with M13 (for/rev) primers (Supplementary Table S1) targeting the pWM321 as well as the pRS1270 monthly (Figures 1C and 2B). Each batch of minimal medium was tested by inoculation of 5 ml complemented medium with *M. mazei* DSM3647. The prepared mitomycin C stock solutions were tested for its ability to inhibit growth of Mm Mut 203b at 2.5 μg/ml with three independent biological replicates. Puromycin stock solution was tested weekly.

### Long-term evolution experiment: determination of excision frequency by a nested qPCR approach

**Figure 3.**
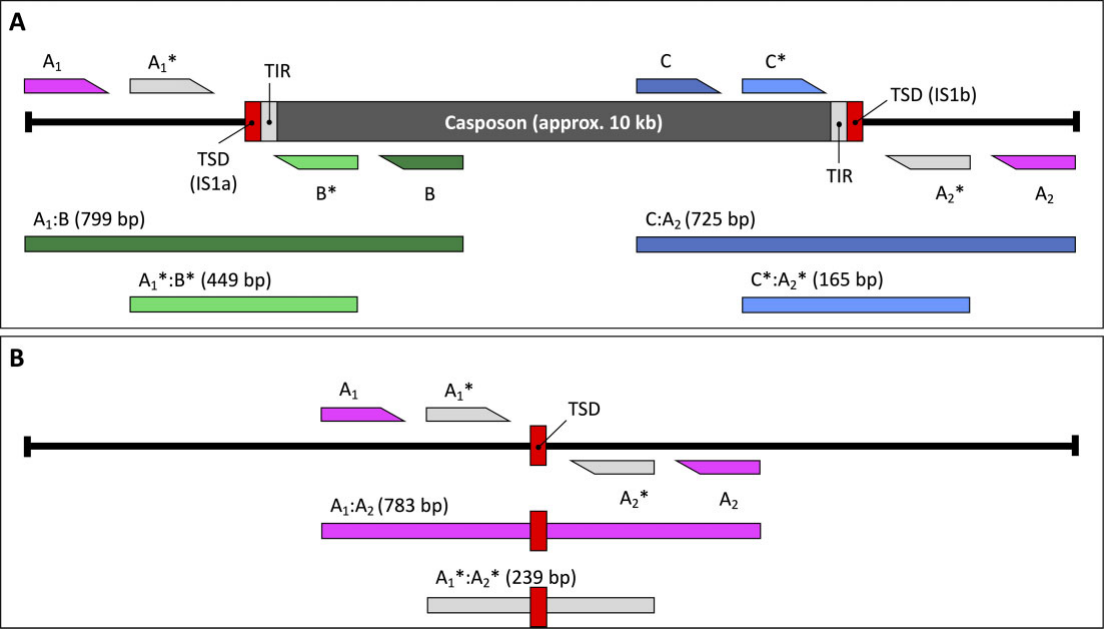
Primer sets used in nested qPCRs, their targets and PCR products. Primer sets were generated by combining one forward and one reverse primer with the same annealing temperature targeting the regions of interest of MetMaz-C1. Primers with an annealing temperature of 47°C (without *) were used in the 1^st^ PCRs, while all primers marked by stars (*) annealed at 55°C were used in the 2^nd^ PCR of the nested qPCR approach. (**A**) MetMaz-C1 is depicted together with the designed oligonucleotides targeting its sequence. Combination of primers A_1_:B (product size: 799 bp) and A_1_*:B* (product size: 449 bp) were used to amplify the left casposon TSD IS1a, while primers C:A_2_ (product size: 725 bp) and C*:A_2_* (product size: 165 bp) were used to amplify the right casposon TSD IS1b. (**B**) Casposon locus post casposon excision leaving an empty target site. 1^st^ PCR of *M. mazei* gDNA using A_1_:A_2_ resulted in a fragment of 783 bp, subsequent 2^nd^ PCR resulted in final product size of approx. 239 bp (A_1_*:A_2_*). These primer sets were used in the nested qPCR approach to determine casposon excision frequency (CEF).

In this study, different PCR protocols were set up for different purposes. The primers were designed to target the native MetMaz-C1 locus in the *M. mazei* genome. Primers belonging to set A were targeting flanking casposon regions, while combinations of set A primers with set B or set C primers were targeting the casposon TSDs IS1a or IS1b (Figure 3A). All primer sets were designed with two different annealing temperatures and target sequences for setting up PCRs or nested qPCRs, since single PCR reactions were found to be not sensitive enough to detect casposon excision events on single cell level. To detect empty target sites as a hint for positive excision and for the determination of the casposon excision frequency (CEF) primer set A was used (Figure 3B).

Selected samples were screened for casposon excision events using a nested qPCR approach (Figures 2B and 3). First PCR reaction was set up using 3 μl gDNA of culture of interest or dilution of pRS1511 control plasmid. The protocol of the 1^st^ PCR was based on manufacturer protocol of GoTaq Polymerase (Promega), with a final volume of 25 μl and the following PCR protocol: initial denaturation at 95°C for 3 min, cycle denaturation at 95°C for 30s, annealing at 47°C for 15 s, cycle elongation at 72°C for 1 min, final elongation at 72°C for 5 min. The primers were used at final concentrations of 0.4 μM per oligonucleotide. Primer set A_1_:A_2_ was used to target the upstream and downstream region of the known casposon location in the *M. mazei* genome (3946587–3956667) (Figure 3). 5 μl of the 1^st^ PCR product was used as template for the 2^nd^ qPCR with a final volume of 25 μl. Plasmid normalized qPCR reactions were set up using PowerUP SYBR Green Mastermix (Thermo Fisher Scientific, Waltham, MA, USA) as recommended. Primers A_1_* and A_2_* were used at a final primer concentration of 0.2 μM per oligonucleotide. qPCR program was similar to the first PCR protocol, except that the annealing temperature was 55°C for 19 s and cycle elongation was set to 30 s. Both PCR protocols were run for 35 cycles. The qPCR protocol included a melt curve analysis using the following steps: 95°C for 15 s, 60°C for 60 s and 95°C for 15 s with temperature gradients at 1.6°C/s. The quantification of nested qPCRs based on the cycle threshold (ct) values was performed by normalization to a 1:10 serial dilution of previously Qubit measured concentration of pRS1511 control plasmid (1x DNA Broad Range Kit) according to methods described elsewhere (31). pRS1511 insert consisting out of the upstream and downstream casposon regions was targeted with the same primer sets as described for the casposon locus mentioned above (Figures 1D and 3). To compare different biological replicates, strains, or time points, normalization to the pRS1713 control plasmid was performed to quantify *M. mazei* genome numbers (copy numbers) per reaction by qPCR in parallel using the same samples and sample volumes. The qPCR protocol was the same as for the 2^nd^ qPCR described above, but the normalization plasmid and the primers were different. A genome quantification control plasmid (pRS1713) was designed by cloning *MM_RS06290* (bifunctional hexulose-6-phosphate synthase) into pCRII-TOPO according manufacture protocol (Figure 1E). Primers, 1713_for and 1713_rev, were targeting the *MM_RS06290* sequence (Figure 1E). Serial dilution was set up in similar manner as described for pRS1511.

### Statistical analysis of casposon excision frequency (CEF) values determined for different samples

For the CEF quantification within ancestor strains, gDNA of freshly inoculated strains with six technical replicates was used, normalizing to three technical replicates of the serial dilutions of pRS1511 and pRS1713. For statistical analysis a two-tailed t-test was performed in GraphPad Prism [v9.3.1 for Mac, GraphPad Software, San Diego, CA, USA]. For comparisons between the CEF values determined for long-term evolution experiment samples, the same number of technical replicates was used for pRS1511 and pRS1713 dilutions, but with three selected biological replicates for each condition and strain with five technical replicates each. The resulting data was analyzed in GraphPad by performing a two-way ANOVA under consideration of multiple comparisons using Šídák's correction. All samples for quantification within one sample set normalized to one of the plasmids were run on the same qPCR plate, to exclude run and plate effects.

### Sequencing and bioinformatics

Plasmids and PCR products were sequenced via Sanger sequencing at the Institute of Clinical Molecular Biology (IKMB, Kiel, Germany). The Sanger sequencing results were analyzed by BLAST and reference-based alignments using Bowtie2 [v2.4.5] (32) or similar as part of Geneious Prime [v2022.2.2.]. The genomes of Mm Mut 203b and Mm Mut 208 were sequenced by Oxford Nanopore Sequencing on a MinIon device in-house and Illumina sequencing (2 × 250 bp, paired-end) at MicrobesNG (Birmingham, UK). Trimming and filtering of Illumina reads was performed by MicrobesNG using in-house scripts based on Trimmomatic (33), Samtools (34), BedTools (35) and bwa-mem (Li, Heng. (2013); preprint; doi: 10.48550/arXiv.1303.3997). Long reads were filtered and trimmed by using *LongQC* [v1.2.0c] (36). For genome reconstruction, trimmed Oxford Nanopore long reads were assembled using *unicycler* [v0.4.8] (37) in long read only mode and contigs were polished with Illumina short reads by using *bwa* [v0.7.15] (Li, Heng. (2013); preprint; doi: 10.48550/arXiv.1303.3997) and *polypolish* [v0.5.0] (38). The polished genome assemblies were manually adjusted in Geneious prime by circularization, followed by manually adjusting genome start site by mapping of first 100 bp of *M. mazei* NC_003901.1 reference to the respective assembly. Genomes were reverse complemented if necessary. The annotation was performed using NCBI Prokaryotic Genome Annotation Pipeline (PGAP) (39–41).

## RESULTS

We obtained two independent lines of evidence that the *M. mazei* casposon actively translocates in the *M. mazei* genome: (i) by using a mini-casposon approach and (ii) by evaluating the chromosomal position of the casposon in *M. mazei* populations during an evolution experiment. The mini-casposon assay was based on a suicide vector carrying a synthetic mini-casposon with a neomycin resistance cassette. During transformation of *M. mazei* cells, the mini-casposon should be able to translocate to the *M. mazei* chromosome resulting in neomycin resistance. The active translocation was analyzed and verified by a PCR and a rescue-cloning approach, followed by Sanger sequencing.

The second approach was focusing on a potential activity of the native, chromosomal casposon MetMaz-C1. For this purpose, two strains, one empty vector control (Mm Mut 203b) and one Cas1solo overexpression mutant (Mm Mut 208) were cultured under four different conditions (Figure 2). The casposon activity was characterized by its casposon excision frequency (CEF), which was determined by nested qPCRs (Figure 3). The nested qPCRs were designed to amplify empty target sites sensitive enough to detect single excision positive *M. mazei* genomes. The generation of PCR products at empty target sites was based on nested PCRs with very short elongation times, so that PCR fragments were generated only when the target sites were empty, which was due to the excision of the full-length casposon of 10 kb. First focus was given to the ancestor cultures, which were used to inoculate the long-term evolution experiment, followed by the characterization of samples of the long-term evolution experiment. Additionally, generated various PCR products were sequenced to obtain information about the structure of the empty casposon locus and potential new integration sites.

### A mini-casposon was actively translocating into the genome of *M*.*mazei in vivo*

**Figure 4.**
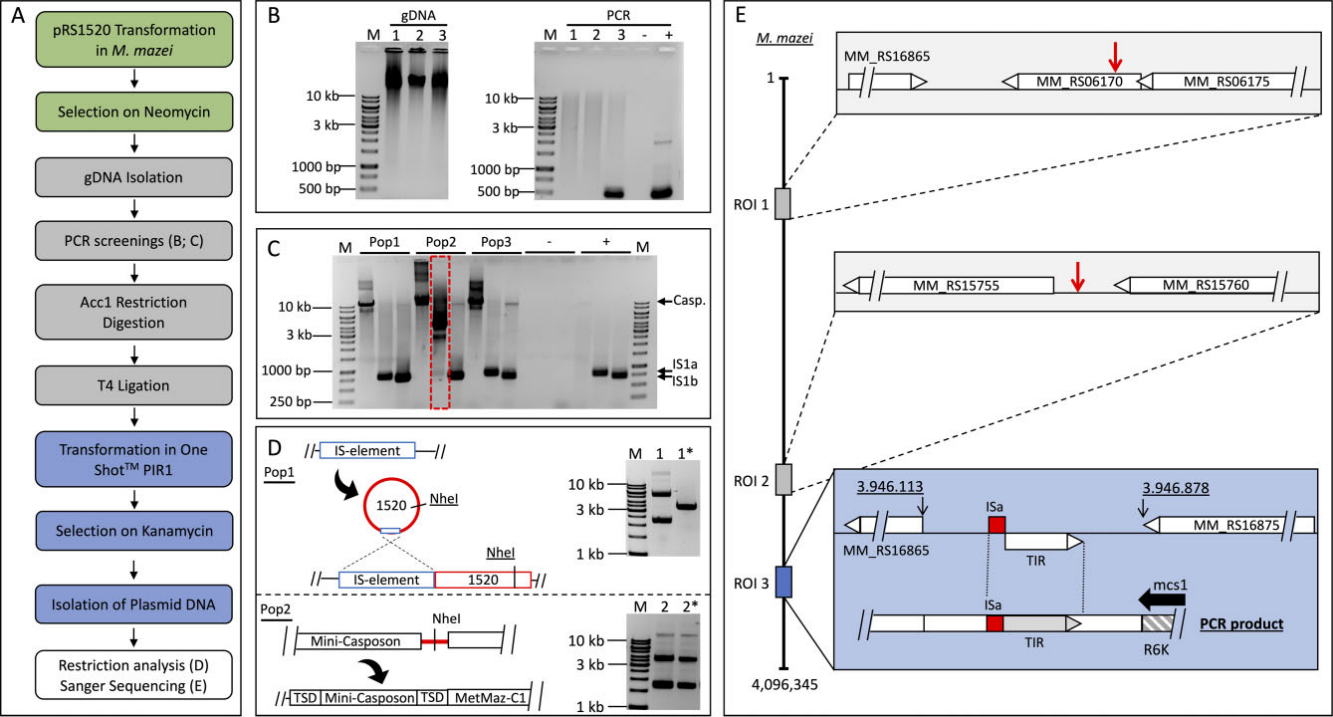
Experiment overview of the *in vivo* mini-casposon assay by PCR analysis of gDNA and rescue cloning. (**A**) Depicted is the pipeline of the mini-casposon assay, which begins with the transformation of *M. mazei* cells with pRS1520. The transformants were selected on neomycin followed by isolation of gDNA. The gDNA was analyzed by PCRs to identify potential positive populations showing mini-casposon activity. Selected gDNAs were restricted by AccI followed by self-ligation with T4 ligase. One Shot™ PIR1 cells were transformed with circularized fragments and selected on kanamycin containing plates. Plasmids of obtained transformants were isolated using Presto™ Mini Plasmid Kit (Geneaid Biotech Ltd., New Taipei City, Taiwan), analyzed by restriction digestion with NheI and sequenced via Sanger sequencing. (**B**) gDNA of three selected populations (Pop 1–3) was used in PCRs with mcs1 and mcs2 primers targeting pRS1520 in comparison to negative water control (-) and pRS1520 (+). **(C)** PCR screening of the three populations (Pop 1–3) for mini-casposon integration into the MetMaz-C1 locus in comparison to negative water control (-) and wildtype gDNA (+).Primer set combinations were targeting the full MetMaz-C1 casposon (A_1_:A_2_), the left target site IS1a (A_1_:B) and the right target site IS1b (C:A_2_) (see Figure 3A). Arrows indicate the expected PCR products. Outlined in red are the differences between populations in the IS1a site, with a roughly 3 kb larger fragment indicating an integration of the mini-casposon into this site. **(D)** Selection of rescue-cloning-derived plasmids of Pop 1 and Pop 2 were treated with NheI (*) or left untreated. Next to the digestion profile the hypothesized mini-casposon integration pattern is depicted. Integration could potentially be connected to IS-element activity (upper part) resulting in a single cross-over or by site-specific mini-casposon mediated translocation (lower part). Single cross-over event is detected by the presence of the NheI site from pRS1520 backbone. **(E)** Verification of NheI digestion results via Sanger sequencing. Reads of rescue-cloning-derived plasmids of Pop 1 and Pop 2 were mapped to *M. mazei* genome obtained from NCBI by Bowtie2 in Geneious Prime. Reads of Pop 1 derived plasmids mapped to two different regions of integration (ROI) 1 and ROI 2, while reads of rescue-cloning-derived plasmids of Pop 2 were mapped to ROI 3 (blue). Integrations into ROI 1 and ROI 2 (gray boxes, red arrows) were found to be in close neighborhood to chromosomal *M. mazei* IS-elements (*MM_RS16865* and *MM_RS15760*). ROI 3 integrations were found to be integrations of the mini-casposon in the left TSD (IS1a) of MetMaz-C1 forming a tandem. The schematic PCR product is showing the binding site of mcs1 primer in the R6K **γ** origin and the following sequence of the mini-casposon TIR and TSD integrated to the IS1a site.

To track a potential casposon translocation *in vivo*, a plasmid-based mini-casposon assay was established. The assay is based on the vector pRS1520, constructed during this study, carrying a mini-casposon (TSD-TIR-R6K **γ** origin-Kan^R^-Neo^R^-TIR-TSD) (Figure 1B). *M. mazei* was transformed with the vector, allowing the mini-casposon if active to translocate from the suicide vector into the host genome using chromosomally expressed essential proteins encoded in MetMaz-C1 (e.g. Cas1solo) (Figure 4A). For detection of positive mini-casposon integrations, *M. mazei* populations were selected for the mini-casposon mediated neomycin resistance (Figure 4A). To rule out potential false positives due to homologous recombination, resistant populations were further characterized with respect to mini-casposon integration as follows. First, to exclude integration of the complete pRS1520 plasmid via single-crossover events, PCRs were used to screen isolated gDNA of neomycin resistant populations for remaining suicide plasmids. For this purpose, PCRs were set up with the two primers targeting the mini-casposon in outside direction (mcs1, mcs2). Samples from populations that generated a PCR product representing an intact suicide vector backbone were excluded from downstream rescue cloning analysis. In these cases, it was very unlikely that the mini-casposon was correctly translocated to the chromosome, but rather the plasmid integrated into the chromosome by a single-crossover. Secondly, selected populations and their gDNA samples were used for the rescue cloning of the casposon with flanking chromosomal DNA fragments, which was based on the mini-casposon mediated R6K **γ** origin and the kanamycin resistance cassette, which both were essential for replication and persistence of rescue-cloning-derived plasmids in *E. coli*. *E. coli* was transformed with the respective circularized chromosomal DNA fragments, selected on kanamycin containing plates and characterized by restriction digestion, PCRs and Sanger sequencing. Sequence analysis of a final selection of plasmids unraveled mini-casposon integration events and sites. The experimental procedure is summarized in Figure 4A.

According to the outlined strategy and analysis pipeline, *M. mazei* wt was successfully transformed with pRS1520 resulting in three neomycin resistant populations (Pop 1–3) (Figure 4B). gDNA of those populations was first analyzed by PCRs using mcs1 and mcs2 primers targeting the mini-casposon ends (outside direction), amplifying the pRS1520 backbone (approx. 500 bp) (Figures 1B and 4B). No PCR product was expected if the mini-casposon had translocated from the suicide plasmid to the chromosome, which was the case for Pop 1 and Pop 2 (Figure 4B). In contrast to this, PCRs with gDNA of Pop 3 resulted in a clear PCR fragment indicating remnants of pRS1520, suggesting a persistent suicide plasmid as extra chromosomal DNA fragment or its integration into the chromosome by a single-crossover. To further distinguish between a directional mini-casposon translocation and a potential integration of the mini-casposon sequence by single-crossovers, the structure of the original MetMaz-C1 locus was analyzed by PCR using the described three primer sets A, B and C (Figure 3A). If the mini-casposon integrated into the original MetMaz-C1 locus, these events should be clearly detectable by variation in the PCR fragment sizes in comparison to the controls. If the mini-casposon would have replaced the whole MetMaz-C1 sequence based on a potential homologous recombination due to TIR and TSD similarity in a double-crossover, the PCR product generated using primer set A_1_:A_2_ would have resulted in a PCR product smaller than the expected 10 kb. This was not the case for any of the tested populations (Figure 4C, first lane for each population). In case that the mini-casposon integrated into one of the original TSDs of MetMaz-C1 generating a tandem structure, changes in size of the PCR products using primer set A_1_:B and C:A_2_ should be visible. Comparisons of PCR fragment sizes of PCRs using gDNA of Pop 1 and 3 as templates with the gDNA control indicated no differences in the target sites, due to equal fragment lengths (Figure 4C, lane 2 in each case). In contrast to these, the PCRs with Pop 2 and primer set A_1_:B were showing a strong difference in fragment size (Figure 4C). PCR fragments were approximately 3 kb larger than the control or the PCR products amplified in Pop 1 and Pop 3 reactions (Figure 4C, Pop2, red box). This suggested that the mini-casposon integrated into the left TSD (IS1a) of the chromosomal MetMaz-C1 in a tandem-like structure (Figure 4D Pop 2).

To verify these results, Pop 1 and Pop 2 were selected for downstream analysis using the outlined rescue cloning approach and the analysis of the presence of the NheI restriction site. After transformation of self-ligation reactions of complete Acc1 digested *M. mazei* gDNA of Pop 1 and Pop 2 into *E. coli*, plasmids of single kanamycin-resistant clones were isolated and verified by restriction digestion with NheI. In case of a positive mini-casposon translocation into the host genome, the NheI site originally present in the pRS1520 backbone cannot be present in the rescue-cloning-derived plasmids (Figures 1B and 4D). Analysis of plasmids derived from Pop 1 showed positive restriction digestion products, due to clear plasmid linearization (Figure 4D). Pop 2 did not show any linearization, which suggested the absence of a NheI site in the rescue-cloning-derived plasmids of Pop 2. These results indicate that a true mini-casposon integration event took place in Pop 2, while Pop 1 seemed to be the result of a single-crossover, although only a small fragment with additional low sequence homology mainly based on the TSDs (2 × 14 nt) and TIRs (2 × 38 nt) between the suicide vector and the *M. mazei* genome was available for homologous recombination. A potential increase of sequence similarity could be achieved by activity of a host derived IS-element during the transformation process (Figure 4D, Pop 1). In case of a translocation of an active IS-element into the fresh introduced plasmid pRS1520, the ability of a homologous recombination of pRS1520 and the *M. mazei* genome would have been increased. These plasmids would be able to integrate into the chromosome by single-crossover as indicated in Figure 4D, Pop 1).

To further verify the obtained results, as well as to address the IS-element hypothesis, rescue-cloning-derived plasmids of Pop 1 and Pop 2 were sequenced via Sanger sequencing using mcs1 and mcs2 primers (Figures 1B and 4E; Supplementary Table S4, mini-casposon integration). Alignments of Sanger sequencing reads to the *M. mazei* reference (NC_003901.1) using Bowtie2 in Geneious prime indicated further differences between rescue-cloning-derived plasmids from Pop 1 and 2. Three different regions of integration (ROI 1–3) were detected (Figure 4E). Reads from Pop 1 showed two regions of integration in the *M. mazei* genome at two different sites (ROI 1 + ROI 2; Figure 4E gray boxes), but further showed remnants of the suicide vector backbone e.g. the NheI site between the TSDs, which was expected based on the restriction results (Figure 4D, E). Sequencing reads directed from the R6K **γ** origin showed breakage after the second TSD and showed similarity to *M. mazei* IS-elements found in ROI 1 and ROI 2 (Figure 4E, gray boxes). These IS-elements belong to the families IS66 (*MM_RS16865*) and IS1634 (*MM_RS15760*) (Figure 4E). The integration close to these IS-elements supported the hypothesis of an integration based on IS-element activity during transformation (Figure 4D, E).

In contrast to the Pop 1 results, the integration into ROI 3 (Figure 4E, blue box), which was detected for rescue-cloning-derived plasmids of Pop 2, showed no remains of the suicide plasmid or unexpected IS-elements. In this case, the mini-casposon integrated into the original left TSD of the MetMaz-C1 casposon (IS1a; ROI 3; Figure 4E). The sequencing reads directed from the R6K **γ** origin showed the TIR and TSD in the expected manner as well as the upstream region of MetMaz-C1 and did not show any remains of the suicide plasmid backbone (Figure 4E). This strongly supports the very first *in vivo* evidence that the mini-casposon successfully integrated into the IS1a site (ROI 3) of the *M. mazei* chromosome in a tandem-like structure (Figure. 4D, E).

### Demonstrating low frequent *in vivo* activity of MetMaz-C1 in untreated ancestor cultures

A long-term evolution experiment was performed with two different *M. mazei* strains under various stress conditions to track casposon movement *in vivo*. The ancestor strains Mm Mut 203b (empty vector control) and the Cas1solo overexpression strain (Mm Mut 208) were sequenced with Illumina and Oxford Nanopore technology prior to the long-term evolution experiment to gain a deeper understanding of native MetMaz-C1 activity. The basal CEF in both strains without any stress treatment was determined by a nested qPCRs approach (see methods section).

**Figure 5.**
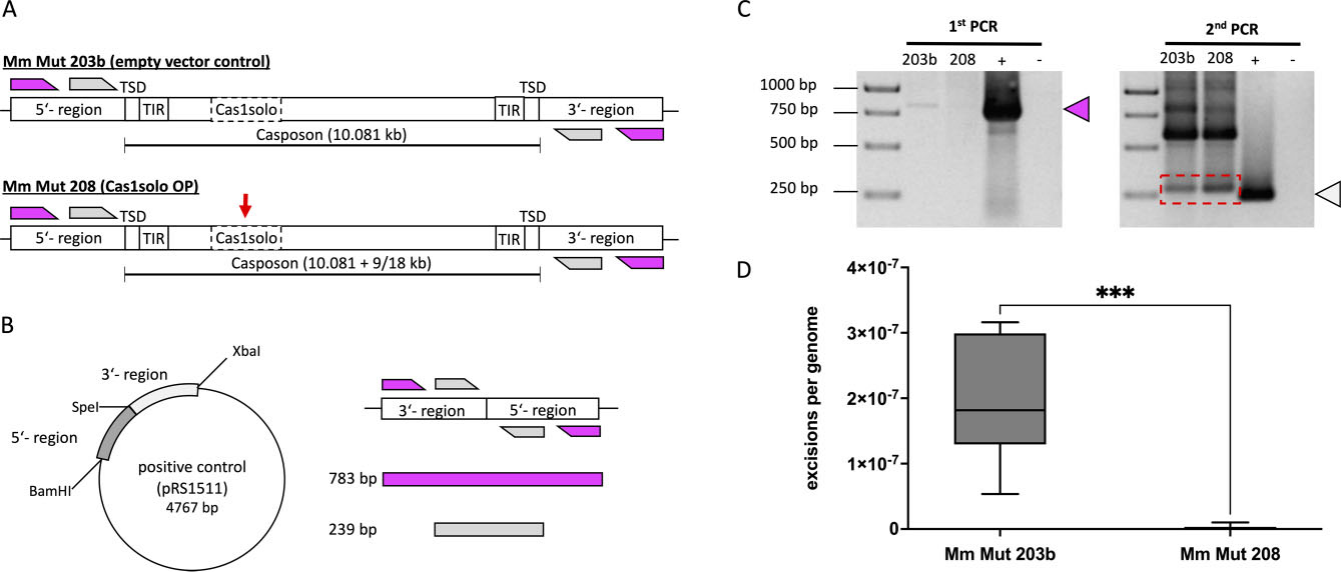
Casposon excision frequency of ancestor strains Mm Mut 203b and Mm Mut 208. (**A**) Mm Mut 203b and Mm Mut 208 were sequenced by Illumina and Oxford Nanopore technology. Hybrid assemblies revealed a high probability of single or double integration of the Cas1solo overexpression plasmid (pRS1270) into the chromosomal *cas1solo* gene of the MetMaz-C1 locus in Mm Mut 208 (red arrow) based on homology of the two *cas1solo* genes. Integration enlarged the casposon sequence by 9–18 kb. (**B**) Control plasmid pRS1511 was designed by cloning MetMaz-C1 flanking regions (upstream (5′-region) + downstream (3′-region)) to pCRII plasmid (see also Figure 1D). PCR products of nested PCRs using pRS1511 as PCR template: 1^st^ PCR reaction = 783 bp and 2^nd^ PCR reaction = 239 bp. **(C)** Analysis of the PCR products of the 1^st^ PCR and 2^nd^ PCR of Mm Mut 203b and Mm Mut 208 in comparison to the positive control plasmid pRS1511 (+) and negative water control (−). Arrow colors refer to PCR products depicted in (B). Red frame highlights nested PCR products. (**D**) Casposon excision frequencies of ancestor strains based on normalization to serial dilution of pRS1511 under consideration of genome copy numbers analyzed by nested qPCRs (*P*-value < 0.001, unpaired, two tailed *t*-test). Quantification was based on measured ct-values (Supplementary Table S3).

Assembled genomes of both strains, based on the described pipeline, showed already high differences in the casposon sequences. Whereas the Mm Mut 203b casposon was unaltered, the Mm Mut 208 assembly indicates a single or double chromosomal integration of the Cas1solo overexpression plasmid (pRS1270) into the host genome. These integration events would increase the size of MetMaz-C1 by 9–18 kb respectively (Figure 5A).

Nested qPCR reactions of gDNA extracted from freshly inoculated strains were set up according to the described procedures in the methods section using the pRS1511-based normalization (Figures 1D and 5B). First nested PCRs indicated empty target sites in a subpopulation of both ancestor strains (Figure 5C). PCR fragments of the expected sizes (783 and 239 bp) in comparison to the pRS1511 control implied empty casposon loci in a subpopulation (Figures 3 and 5B, C). The 1^st^ PCR product with the expected size of 783 bp was detected in low amounts in gDNA of Mm Mut 203b based on a weak signal detected by gel electrophoresis (Figure 5B,C). The 2^nd^ PCR, with a final product size of 239 bp, highly increased signals of empty casposon sites found in both ancestor strains (Figure 5C, right panel red box). Quantification of the CEF based on determined ct-values of the nested qPCRs relative to the quantification of genomes (see methods section), led to deeper characterization of the strains (Figure 5D; Supplementary Table S3). Although PCR products were detected in both strains at the end of the nested qPCR procedure, the calculated CEFs were highly different (Figure 5D). Unexpectedly, the empty vector control Mm Mut 203b showed a much higher frequency (mean: 1.97 × 10^−7^ ± 0.97 × 10^−7^ excisions per genome) compared to a mean of 0.19 × 10^−8^ ± 0.40 × 10^−8^ excisions per genome determined for the Cas1solo OP Mm Mut 208. This difference between the two different strains was highly significant based on a two-tailed t-test (Figure 5D; *P*-value = 0.0001; Supplementary Table S3). One possible explanation for the difference in CEF might be the length of the casposon (see discussion). Overall, the results strongly argue for active MetMaz-C1 excision in *M. mazei* subpopulations *in vivo*.

### Verification of casposon excision and integration into new genomic loci *in vivo*via sanger sequencing

**Figure 6.**
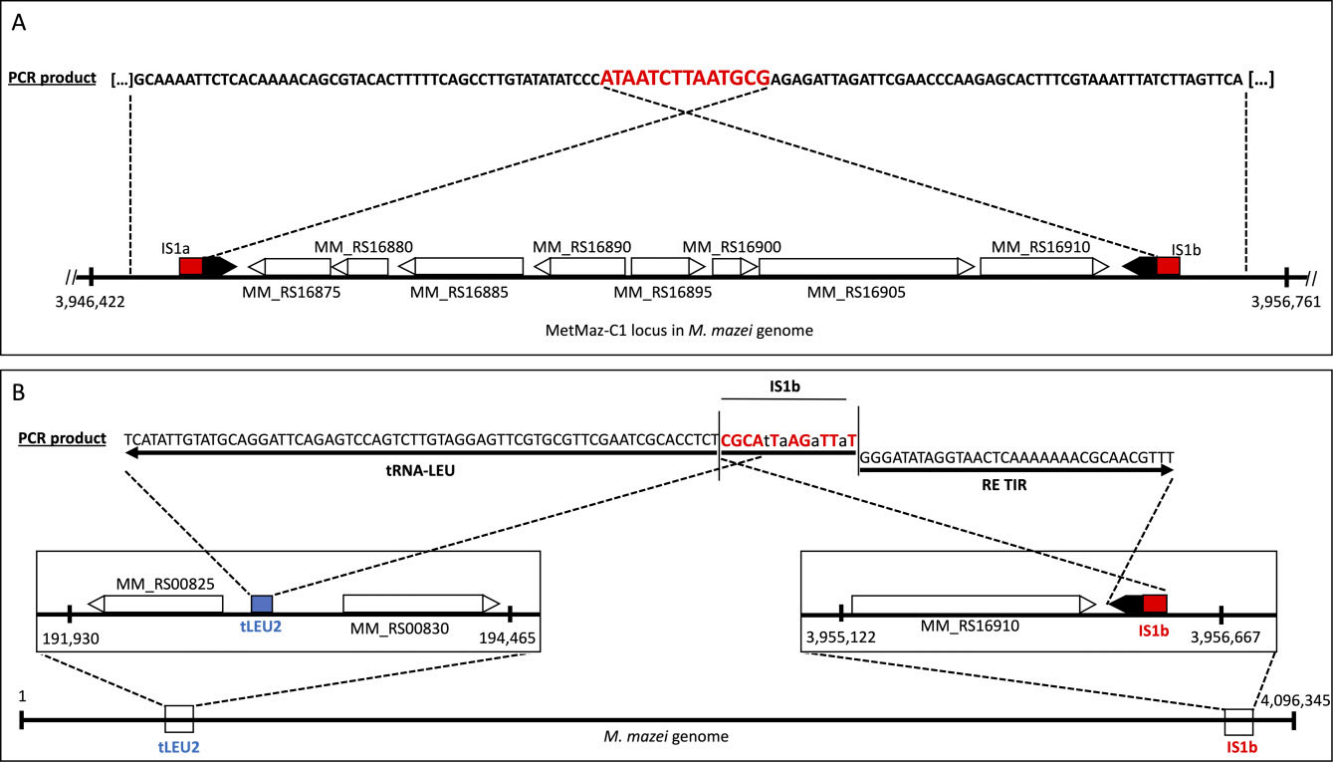
Sanger sequencing of nested PCR products compared to the *M. mazei* NC_003901.1 reference. (**A**) Nested PCR products (primer set A*; Figure 3) were mapped to *M. mazei* reference. Excision of MetMaz-C1 from its original locus (3946587 - 3956667) was observed by leaving a single target site (red boxes), without detectable footprints or remains of TIRs (black arrows). (**B**) Mapping one example PCR product derived by amplification of tLEU2 site (193177 – 193190; CGCAcTtAatTTtT; blue) with site-specific primers and casposon targeting primers C* (Figure 3). PCR product indicated integration of MetMaz-C1 TIR and TSD into tLEU2 site. Integration resulted in replacement of the original tLEU2 site with the reverse complement of the right MetMaz-C1 TSD (IS1b; AtAAtCTtAaTGCG). Identical nucleotides of the two different target sites are indicated by upper case and red color. All PCR product sequences can be found in Supplementary Table S4.

For verification of the casposon excision by the nested qPCR approach, the resulting nested PCR products of both strains obtained above were sequenced via Sanger sequencing using the qPCR primers A* (Figure 3B). Sequence alignments to the reference genome revealed empty target sites, without any casposon footprints or TIRs (Figure 6A). The native casposon MetMaz-C1 was cut out of the genome sequence by leaving a single empty target site (Figure 6A). These results were verified by sequencing PCR products generated from 85 cultures of the first culture plate of the long-term evolution experiment (Supplementary Table S4). Based on this finding *M. mazei* genome loci were checked for potential new integration of the native casposon after 5–6 generations. Potential insertion sites with similarity to already published potential insertion sites (19) were tested by additional nested PCR reactions. Primers B and B* or C and C* (Figure 3A) were used in combination with primers upstream the locus of interest (Supplementary Table S1). These nested PCRs revealed the presence of MetMaz-C1 TIRs and TSDs in at least one new site which was previously published by Krupovic and colleagues (19); Figure 6B; Supplementary Table S4). One example site designated ‘tLEU2’ (193177 – 193190), due to its partial similarity to a tRNA-LEU gene in the *M. mazei* genome was found to contain the MetMaz-C1 TSD and TIR sequence. The PCR fragment derived from site-specific primers and primer set C (Figure 3A) displayed the sequence of the right TIR and the TSD (IS1b) followed by the sequence of the tRNA-LEU homologous region (Figure 6B, Supplementary Table S4). The sequence did not show the original tLEU2 site (CGCAcTtAttTTtT), but a reverse complement the original right TSD of MetMaz-C1 (AtAAtCTtAaTGCG; Figure 6B), indicating a potential integration of MetMaz-C1 by transferring at least one of its own target sites (Supplementary Table S4), which somehow replaced the original tLEU2 site of the locus with IS1b (AtAAtCTtAaTGCG). At least one further example was obtained with an additional tRNA-Leu integration site (tLEU1; CGCATCAAATTTCT; 181365–181378; see Supplementary Table S4). These findings suggest a different translocation mechanism than the mechanism originally proposed by Krupovic (17).

### *In vivo* casposon activity in the long-term evolution experiment influenced by strain and treatments

**Figure 7.**
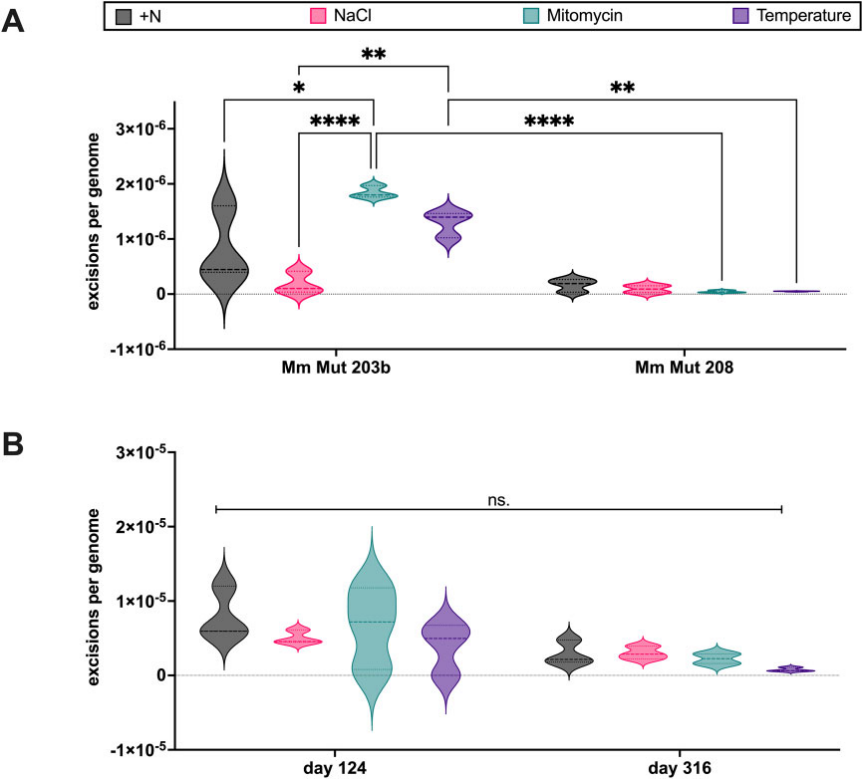
*In vivo* casposon excision frequency in long-term cultured *M. mazei* strains. Excision frequency was determined by nested qPCRs with normalization to genome copy numbers based on qPCRs. Data from three biological replicates, each with five technical replicates, are shown. Datasets were analyzed by two-way ANOVA (*P*-values: ≤ 0.05 = *, ≤ 0.01 = **, ≤ 0.001 = ***, ≤ 0. 0001 = ****). (**A**) Influence of treatments on casposon excision frequency after the first incubation period of 4 days in the defined treatments. gDNA of Mm Mut 203b and Mm Mut 208 was analyzed by nested qPCRs for casposon excision events. (**B**) Effect of long-term cultivation on casposon excision frequency. Casposon excision events in Mm Mut 203b cultures under the defined treatments were determined at two different time points from the long-term evolution experiment.

Due to the influence of cellular stress on transposon activity (42–47), we assumed that stress might also enhance casposon activity. Consequently, the effect on the CEF of four different stress conditions (500 mM NaCl; 0.5 μg/ml mitomycin c; 40°C; +N (N-sufficiency)) was investigated using 12 biological replicates each of the two *M. mazei* strains Mm Mut 203b and Mm Mut 208 in a long-term evolution experiment. Estimation of the CEF based on the previously described nested qPCR approach, was established for comparison of casposon activity in both strains and all used treatments. Different time points of the long-term evolution experiment were selected to determine the respective CEF. Based on the assumption that selection pressure is highest at the first contact of an organism with a changed environment and that this pressure decreases over time by adaptation to this stress condition, the first samples of the long-term experiment was considered first to investigate the effects of different conditions on *M. mazei*, especially on the activity of transposons or transposon-like elements such as MetMaz-C1. The first sample of the experiment was taken after 4 days of incubation of the two strains under the four different conditions. For monitoring the MetMaz-C1 activity based on excision and integration, gDNA of these samples was isolated and analyzed by nested qPCRs (Figure 7A; Supplementary Table S4). Quantitative analysis confirmed the results above and showed statistically significant differences of the CEF between the two strains used, moreover it demonstrated differences between the treatments (Figure 7A).

The CEF values between the two strains cultured under the four conditions still showed the same trend as the ancestors, yet the four cultivation conditions had a large effect on the CEF value determined within the treatments of strain Mm Mut 203b. While the CEF in Mm Mut 203b cultures, grown under normal growth conditions (Mm Mut 203b:+N), varied in a broader range, the frequencies of the other three treatments were rather narrow and significantly different from each other. Cells grown under high salt stress (Mm Mut 203b:NaCl) showed the lowest excision frequencies, whereas cells grown under mitomycin C challenge (Mm Mut 203b:mitomycin) showed the highest. Frequencies measured under high temperature stress (Mm Mut 203b:40°C) were between the frequencies of the other two treatments (Figure 7A). The differences between these three treatments were statistically significant based on two-way ANOVA. The quantitative analysis of Mm Mut 208 cultures treated with the four different conditions did not show any significant differences among each other, but in comparison to the corresponding Mm Mut 203b cultures in case of the mitomycin and the temperature treatment (Figure 7A; Supplementary Table S5).

To follow the CEF under evolutionary adaptation to the treatments, two time points of the long-term evolution experiment were selected and analyzed with the same nested qPCR approach. For this analysis only Mm Mut 203b samples were used because of the detected influence of treatments on the CEF in this strain Figure 7A. CEF values were determined for samples taken at day 124 in comparison to samples taken at day 316. This time period of 192 days represented roughly 250 generations of stable growth. The comparisons of treatments and time points did not show any significant differences, but a trend to a lower variation of CEF values in long-term cultures was observed. The enrichment of casposon excisions over time was not observed during the long-term evolution experiment, consistent with the expectation of highest selection pressure at the beginning of each treatment (Figure 7B; Supplementary Table S6).

## DISCUSSION

Casposons, described as a new class of transposons in 2014, have been found in several archaea species such as *Aciduliprofundum boonei* and some *M. mazei* strains, but their activity has been analyzed or predicted exclusively by computational analysis or a few *in vitro* studies using recombinant Cas1solo protein (17,19,22,25). Demonstration of casposon activity in *in vivo* assays was so far not possible to detect, due to the low number of available model organisms, which are genetically tractable. Therefore, the behavior of the native *M. mazei* casposon MetMaz-C1 was characterized *in vivo* in the current study using two different experimental approaches: (i) determination of excision events of the native casposon analyzed by nested qPCRs of *M. mazei* cultured under different conditions, and (ii) a genetic approach using a mini-casposon clearly demonstrating excision and integration of the casposon *in vivo*.

For characterization of the casposon translocation mechanism, the key processes of excision and integration are discussed individually, starting with the process of casposon excision. During the characterization of the long-term evolution experiment samples and its ancestor cultures by nested qPCRs, deeper insights into the excision mechanism of the *M. mazei* casposon MetMaz-C1 were gained. In this study, the CEF was established as a key marker for casposon activity *in vivo*, allowing to detect casposon excision on a very low level with 10^−5^–10^−9^ excision events per genome. These low values obtained for excision and the necessity of nested PCR approach suggested casposon activity on a very low level. Only single cells of the observed populations or even single chromosomes of their multiple genome copies showed casposon activity. CEF values determined for the empty vector control strain (Mm Mut 203b) and for the Cas1solo overexpression strain (Mm Mut 208) were significantly different to each other. Unexpectedly, the CEF was significantly higher in Mm Mut 203b than in Mm Mut 208. This strong significant difference between the two strains could have various reasons, a simple explanation might be a casposon size effect. The observed size difference of the two casposon variants of Mm Mut 203b and Mm Mut 208 of 9–18 kb resulted from a single or double integration event of the Cas1solo overexpression plasmid into the *M. mazei* chromosome. Based on the 100% identical *cas1solo* genes, encoded on the overexpression plasmid pRS1270 and on the chromosome in the casposon locus, a homologous recombination between these two genes was highly likely. For *M. mazei*, homologous recombination was shown to depend on similarity between regions of at least 800 bp (27). One potential indication for a size dependent excision might be identified by comparing CEF values of Mm Mut 203b with reported excision frequency values determined for other IS-elements. A comparable qPCR approach using Taqman probes was established for quantification of excision frequencies of the IS-element IS492 found in *Pseudoalteromonas atlantica* (48). Determined excision frequencies of IS492 were at least 100–1000-fold higher compared to the frequencies determined for the two MetMaz-C1 variants in Mm Mut 203b and Mm Mut 208 in the current study. Both transposable elements differ significantly in size, the native MetMaz-C1 is roughly eight times larger than IS492 (1200 bp) (19,49), which might have a significant impact on its translocation frequency. Additionally, a comparable size effect was found for the transposon Tn10, where smaller variants were excising more frequently (50). A further explanation might be the genomic environment of MetMaz-C1, which could have inhibitory impact on the excision frequency as discussed for IS492 (48). Alternatively, a decreased CEF in Mm Mut 208 might be based on the strong plasmid derived constitutive expression of Cas1solo, since this kind of expression was shown to frequently cause inhibitory effects on transposable elements (reviewed in (51)).

An alternative explanation for detected low level CEF values of both strains in comparison to IS492 might be a direct reintegration of MetMaz-C1 into the same genome locus. These kind of event would not be detectable with the nested qPCR approach used in this work, which was only targeting empty target sites based on the time point of sampling, and thus might result in underestimation of the CEF. For the IS-element IS492 it was shown that excision events were not crucially coupled with insertion into new sites (48,52). Therefore, it is necessary to mention that this kind of explanation of direct reintegration into the same locus might be ruled out for MetMaz-C1 by qRT-PCRs targeting the casposon genes, especially the *cas1solo* gene encoding the key enzyme, the casposase. c*as1solo* transcription was slightly higher under *Methanosarcina* spherical virus (MetSV) challenge, but showed low expression values in untreated cells, which might explain a very low basal casposon activity (31). Additional stress conditions used in the long-term evolution experiment had little effect on the CEF, in contrast to reports of several organisms in which transposon activity was increased by stress treatment. In this study mitomycin C treatment was chosen based on its direct inhibition of DNA synthesis in bacteria (53), since its ability to crosslink DNA molecules mediating DNA damage (reviewed in (54)). Mitomycin C was further shown to positively influence recombination frequency in *Drosophila melanogaster* and induces the lambda prophage (55,56). The CEF in Mm Mut 203b:mitomycin was significantly higher in comparison to the corresponding untreated Mm Mut 203b:+N. This observation might be interpreted that casposon excision is a recombination-related process since an increase in recombination frequency was observed in *Drosophila* under mitomycin influence. Further, the comparisons of Mm Mut 203b:NaCl to Mm Mut 203b:mitomycin and Mm Mut 203b:40°C were significant as well. NaCl treatment was used in the experimental design to allow a higher expression of the Cas1solo, which has been already shown for CRISPR locus associated proteins in *M. mazei* (57), and thus to positively influence the casposon activity in the same way. Low Mm Mut 203b:NaCl CEF values, not significant in the comparison to untreated cells (Mm Mut 203b:+N), might potentially imply a higher genome stability in the casposon region during high salt stress. Moreover, Cas1solo overproduction did not increase casposon activity in Mm Mut 208 (Figure 7). Therefore, an effect of NaCl stress on Cas1solo-mediated casposon activity seemed to be very unlikely or was completely masked by the casposon size effects. Variation of cultivation temperature was further shown to affect transposon activity in many different organisms but had only a slight effect on casposon activity in the current study. Tn3, characterized in *E. coli* C600, or transposons found in the archaeon *Halobacterium halobium* can serve as examples for cold stress induced transposons, whereas many transposons found in plants such as *Arabidopsis sp*. showed heat induction (44,46,47).

**Figure 8.**
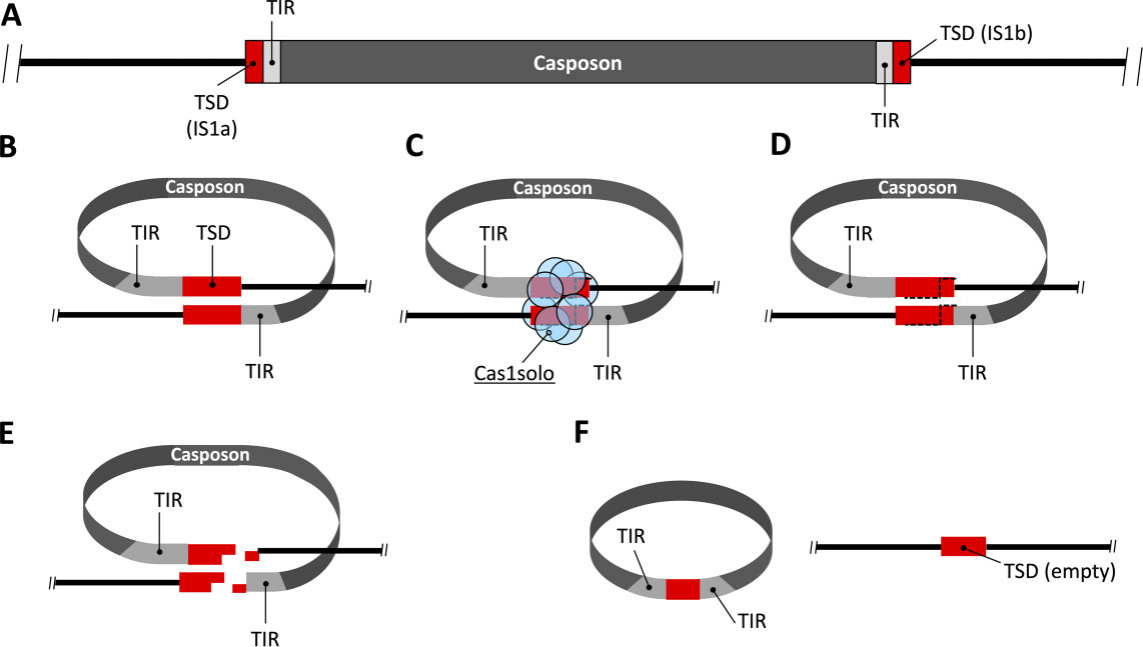
Hypothetical model of casposon excision. (**A**) MetMaz-C1 in the original casposon locus in the *M. mazei* genome (3946587–3956667) with its target sites (TSD; red) and terminal inverted repeats (TIR; light gray). (**B**) Loop-structure essential for casposon excision from its original target site. Target sites are brought close to each other, potentially coordinated by Cas1solo oligomers (**C**). (**D**, **E**) Cas1solo is introducing a staggered cut within the target sites. (**F**) Religation of the digestion fragments lead to one empty target site left in the genome and a circular intermediate of the casposon.

Sequencing of PCR products revealed that only a single empty target site remained in the genome after casposon excision (Figures 6 and 8; Supplementary Table S4). This finding is in disagreement with the model proposed by Krupovic and colleagues (17), which predicted a persistent duplication of the target site, potentially separated by common transposon footprints (17). However, for the MetMaz-C1 excision, a model close to the translocation of IS492 is more likely (48,49). IS492 was shown to generate a single precise empty target site lacking persistent duplication post excision and forming a circular transposition intermediate (48,49). Consequently, based on our findings we hypothesize a circular MetMaz-C1 intermediate during the excision process (Figure 8), although we were not able to detect it during the current study. However, based on the overall very low CEF values determined for MetMaz-C1 compared to the frequencies determined for IS492 (48), it is hardly surprising that this intermediate was not detected. In case of IS492 (with high excision frequency) the circular intermediate was not persistent over extended periods of time either and could only be detected in special treated cultures at low amounts (49). Leaving one single empty target site during casposon translocation further suggests a staggered cut within the target site (Figure 8), like it was first discussed by Krupovic and colleagues (17). Studies of *Polinton/Maverick* transposons carrying capsid proteins in rare cases and descriptions of virus encoded Cas genes (58,59), combined with a potential circular casposon intermediate could be a potential hint for a plasmid or circular virus derived origin of casposons.

To detect potential reintegration into predicted target sites (17,19), additional nested PCRs of these sites were performed (Figure 6; Supplementary Table S4). Integration of the native casposon in the described tLEU2 site (CGCAcTtAttTTtT) was detected via Sanger sequencing of PCR products. PCR products suggested integration in the tLEU2 locus by inversion of the casposon sequence and integrating the original target site. This kind of integration is the second hint to a circular intermediate carrying its own target site during translocation (Figure 8). Integration by homologous recombination is very unlikely, due to very short overlap of sequences by the described GCGA-motif (17,19,22). The process of this ‘replacement’ of the original tLEU2 site is unknown and may also be a kind of artifact, because all our analyses were based on nested PCRs with gDNA of whole populations. Therefore, it could not be excluded that the casposon locus in question, which served as the basis for the PCR product discussed here and was possibly present in this form in only one genome, already carried a mutated version of the tLEU2 site before a potential casposon integration.

Casposon activity *in vivo*, its excision and integration, was further verified during the second genetic approach, the plasmid based mini-casposon assay. Many preceding studies focusing on MGEs used plasmid-based transposition assays to characterize transposons or integrating phages e.g. Mu-phage (60). Many of these systems use at least two separated plasmids, where one plasmid carries the engineered transposon and helper plasmids carry transposition-dependent genes e.g. transposases. These systems were often optimized for generation of mutant strains by introduction of genes of interest into target genomes (61). The *in vivo* mini-casposon assay used in the current study is completely independent from other components, since its potentially relevant proteins are chromosomally expressed by the MetMaz-C1 locus (e.g. Cas1solo). A cross element/cross system activity between the casposon and the *M. mazei* CRISPR-Cas systems regarding Cas1 was unlikely, since *M. mazei* CRISPR-Cas systems were found to be inactive (57). The mini-casposon designed on the suicide vector pRS1520 consisted of MetMaz-C1 derived TSDs and TIRs flanking a kanamycin resistance cassette, a neomycin resistant cassette and a R6K **γ** origin, and was therefore in analogy of various preceding studies with transposons (23–25,62–64). If this engineered casposon was active, it would result in integration of the mini-casposon into the *M. mazei* genome at its specific target sites mediating a neomycin resistance. Analysis of neomycin-resistant *M. mazei* populations by PCRs and a rescue cloning approach adapted from Quin and colleagues (62), lead to the conclusion that the mini-casposon had actively integrated into the host genome. Sequencing of positive rescue-cloning-derived plasmids showed correct mini-casposon translocation to the left TSD (IS1a) of MetMaz-C1 in at least one case. Further incorrect integration events of the suicide plasmids based on single-crossover events were observed in close association with *M. mazei* derived IS-elements, which imply activity of these elements as well. We hypothesize IS-element translocation to the freshly introduced suicide plasmid (pRS1520) by accident, followed by homologous recombination into another copy of the host chromosome via the IS-element (see Figure 4D).

The obtained results and arguments discussed within this study allowed first deeper insight in the casposon activity *in vivo*. Data suggested low level casposon activity in single cells of a *M. mazei* population, which is neither beneficial nor lethal in most cases for the respective host cells and thus will not be enriched. MetMaz-C1 and the used mini-casposon showed strong site-specific integration as it was already concluded for different casposons by characterization of their key enzymes *in vitro* (22–24). MetMaz-C1 appeared to excise and integrate by potentially transferring the original target site, suggesting a staggered cut within the target sites (Figure 8). Based on our data, we hypothesize, that the casposon is forming a circular intermediate, although we were not able to detect such an intermediate (Figure 8). The mini-casposon assay revealed integration in the same MetMaz-C1 target site resulting in tandem structures, which potentially counts as a further argument for its evolutionary role in the generation of CRISPR like arrays. Such tandem structures were reported for a huge variety of IS-elements, transposons and even for some casposon relatives in different *Methanosarcina* species (reviewed in (6,19). Overall, the current study not only gave first experimental evidence for *in vivo* activity of MetMaz-C1 but in addition also indications for revising the previous translocation model.

## Supplementary Material

gkad474_Supplemental_Files

## Data Availability

Assemblies of Mm Mut 203b and Mm Mut 208 as well as belonging raw data are provided under the following Bioproject IDs: PRJNA929716 and PRJNA929891. Supplementary Table S1: List of all used primers. Supplementary Table S2: Growth data of *M. mazei* cultivated in deep-well plates. Supplementary Table S3: Summary of CEF values determined for ancestor strains. Supplementary Table S4: Sanger sequencing reads of PCR products. (can further be provided in original *.fastq* or *.ab1* files by request). Supplementary Table S5: CEF values determined for strains treated with different stress conditions; Supplementary Table S6: CEF comparison of long-term evolution experiment time points.
