# Supplementary material for: Active in vivo translocation of the Methanosarcina mazei Gö1 Casposon
Source: Nucleic Acids Res. 2023 May 31;51(13):6927–43. doi: 10.1093/nar/gkad474 (PMC10359463; doi:10.1093/nar/gkad474)
Supplement: gkad474_Supplemental_Files [file gkad474_supplemental_files.zip › supplementary_file_legends_NAR-00320-H-2023.R2_Gehlert.docx]

**supplementary file legends**

**Table_S1: List of all used primers.**

**Table_S2: Growth data of *M. mazei* cultivated in deep-well plates.** Optical densities measured at 600 nm (OD600) of *Methanosarcina mazei* cultured under three different conditions (mitomycin C; NaCl; +N). 12 replicates of *M. mazei* were grown under the defined treatments. Subsamples were taken after defined time points. Growth rate was calculated by the formula: Growth rate [gr] = ln(OD_end_/OD_start_)/(t_end_-t_start_). The doubling time was calculated with the formula: Doubling time [dt] = ln(2)/gr.

**Table_S3: Summary of CEF values determined for ancestor strains.**

**Table_S4: Sanger sequencing reads of PCR products.** Table summarizes all sequenced PCR products. "Bold" sequences were used as examples in the main text. Data sheet "excision" summarizes all sequences used for determination of the casposon excision. All sequences are representing independent biological replicates from the long-term evolution experiment. Data sheet "integration" gives a summary of sequenced PCR products of potential integration sites within the *Methanosarcina mazei* genome. Data sheet "mini-casposon integration" lists PCR products generated from rescue-cloning derived plasmids of Pop 1 and Pop 2.

**Table_S5: CEF values of determined for strains treated with different stress conditions.**

**Table_S6: CEF comparison of long-term evolution experiment time points.**
